# Miscellaneous

**Published:** 1896-11

**Authors:** 


### MISCELLANEOUS.

### EXTRACTS FROM NOTES ON APENTA AND OTHER WATERS.

BY JULIUS ALTHUS, M.D.,

Consulting Physician to the Hospital for Epilepsy and Paralysis, Regent's Park.

The individual differences which are found to exist in the chemical composition of mineral waters belonging to the same group are of considerable interest from a pharmacological as well as therapeutical point of view. Mineral waters generally contain one or at most two principal ingredients, in addition to which, however, there are habitually two or three other substances present which modify the effects of the prime constituents in a peculiar manner. These "modifiers," as I would call them, oftener occur in very small quantities, and have for this reason by many been considered as inoperative. Such an opinion, however, is erroneous, for careful clinical observation of the action of such waters shows the "modifiers" to be of considerable importance, so that a mineral water would find its individuality impaired or destroyed, were they to be removed. I will remark in passing that this is the principal reason why artificial mineral waters in which the "modifiers" are often left out, in order to save trouble in the manufacture, have not the same therapeutical effects as natural waters.*

Apenta belongs to the class of bitter waters, and contains, as its principal ingredients, the sulphates of magnesium and sodium, while its chief modifiers are the chlorides of sodium and lithium. The medicinal value of bitter waters in general depends to a great extent upon the relative proportion of the two sulphates contained in them.

Pharmacological experiments and clinical experience have shown that those waters act most pleasantly in which the magnesium preponderates over the sodium sulphate, while the chloride of sodium should not amount to more than about $\frac{1}{10}$ or $\frac{1}{20}$ of the two sulphates together. Where the sodium is in excess of the magnesium sulphate, the purgative action of the bitter water is *cœteris paribus* too violent; digestion is apt to be disturbed, and if the use of the water is continued for some time, debility and a degree of anemia will be the result. On the other hand, where the chloride of sodium is in excess, the water does not act so beneficially on the liver.

If we apply the above rules to some of the best known bitter waters we find that:

. . . . . . . . . . .

Apenta water shows an excellent proportion of sulphates and chlorides, the magnesium amounting to 24.49, the sodium sulphate to 15.43, and the NaCl to 1.87. It is therefore a most useful

*I have dwelt fully upon this point in my work on "The Spas of Europe," London 1862.

aperient. In addition to this it contains an appreciable amount of lithium chloride, by which it is distinguished from all the other waters of this group. Although the quantity of this constituent is small, it is for that reason by no means devoid of effect as a "modifier."

I may here aptly draw attention to the very small quantity of iron contained in such powerful chalybeates as Spa and Schwalbach (Pouhon 0.375, and Weinbrunnen 0.443 grains in 16 ounces of water). Again, the Kreuznach Elisenquelle, which is so highly effectual in the treatment of scrofula and allied conditions, contains only 0.035 grains of magnesium iodide in 16 ounces, and the Purton water only 0.066 of sodium iodide in the imperial gallon. The presence of lithium in Apenta water explains, therefore, why a course of the latter is so useful in warding off attacks of gout, and in moderating their intensity when present.

It is useful to know that in cases where we are obliged to give morphine in somewhat considerable quantities, either *per os* or hypodermically, as for instance in the gastric crises of tabes, and in severe and obstinate forms of neuralgia, Apenta has an excellent effect in neutralising the prejudicial action of morphine on the hepatic functions. In some of these cases I had previously given small doses of mercury, podophyllin, and other drugs and mineral waters for that purpose, but none of them equalled the beneficial effects of Apenta in this respect.

The same water is useful, as an adjuvant to other treatment, in threatened cerebral hemorrhage in the aged, where there is high tension in the vascular system, with confusion, drowsiness, etc. On the other hand, it sometimes fails to relieve obstinate constipation which arises from deficient nervous influence, as in certain forms of spinal disease, where more stimulating aperients, such as cascara sagrada, etc., find their appropriate sphere of action.—*British Medical Journal*, 26th September, 1896.

---

### HOW TO WRITE A MEDICAL PAPER.

The New York *Polyclinic* (March 15) contains some good suggestions and ideas on this important and always appropriate subject. The writer says that comparatively few who write for publication in medical journals succeed in placing their material before their readers in a form that is to add to the general store of knowledge. One is too often impressed by the array of minutely detailed histories, repetition of well-known facts, and the like, which are entirely unnecessary to the proper elucidation of the subject, and the perusal of which merely takes up the reader's time without a commensurate benefit accruing therefrom. In reporting an experience with a certain therapeutic measure, it is entirely unnecessary, in a majority of instances, to publish a long dissertation upon the history of the particular method under discussion.

A useful preface to a treatise, or report of a case, is one that states in a few words the author's purpose in presenting it. Medical men, in their hurried reading, do not care for a long-drawn-out preface, nor do they attach as much importance to a minutely-detailed history of a case as they once did; they prefer only the salient points which bear directly upon the purpose of the article.

Medical men are busy men, and therein often lies the reason, although not a good one, for the careless preparation of an article which might be excellent were it not for the unfortunate characteristic mentioned. The value of their literary contributions may often be greatly enhanced by a careful and painstaking editor, who arranges them, cuts out irrelevant matter, and in general brings them from literary chaos to a condition which, in a measure, fits them to meet the expectations of the author and satisfy the requirements of the reader. Although many of our professional brethren have not had the advantages of a higher literary education, still, few of them are really ignorant, and there is no legitimate reason why all, when necessity or inclination prompts, should not wield that mightiest of weapons, the pen, to the best advantage.

1. In your writing be, above all things, *purposeful;* afterward, concise, relevant, definite.

2. The subject selected should contain but one definite line of thought; should not be trite; should be one in which you are personally interested; if argumentative, one in which you have convictions. It should be suited to your abilities and to your opportunities for forming a correct judgment of the merits of the subject treated; should be stated intelligently; should be no broader than the essay itself.

3. Make an outline of your subject; it will enable you to read up accurately and profitably; it will afford mental discipline.

4. Be careful to pay attention to the elegance of your language; to such little things as correct paragraphing and punctuation.

---

### Relations of Medical Examining Boards to the State, to the Schools, and to Each Other.

Dr. William Warren Potter, of Buffalo, president of the National Confederation of State Medical Examining and Licensing Boards, chose this title as the subject of his annual address at the sixth conference of this body, held at Atlanta, May 4, 1896.

He said there were three conditions in medical educational reform on which all progressive physicians could agree—namely, first, there must be a better standard of preliminaries for entrance to the study of medicine; second, that four years is little enough time for medical collegiate training; and, third, that separate examination by a State board of examiners, none of whom is a teacher in a medical college, is a prerequisite for license to practice medicine. It is understood that such examination can be accorded only to a candidate presenting a diploma from a legally registered school.

He further stated that a high school course ought to represent a minimum of academic acquirements, and that an entrance examination should be provided by the State for those not presenting a high school diploma or its equivalent.

He did not favor a national examining board as has been proposed, but instead thought all the States should be encouraged to establish a common minimum level of requirements, below which a physician should not be permitted to practice; then a State license would possess equal value in all the States.

In regard to reciprocity of licensure, Dr. Potter thought it pertinent for those States having equal standards in all repects to agree to this exchange of interstate courtesy by official indorsement of licenses, but that other questions were of greater moment just now than reciprocity. Until all standards were equalized and the lowest carried up to the level of the highest, reciprocity would be manifestly unfair.

He urged that the States employ in their medical public offices none but licensed physicians. This, he affirmed, would tend to stimulate a pride in the State license, and strengthen the hands of the boards.

He denied that there was antagonism between the schools and the boards, as had been asserted. He said that both were working on parallel lines to accomplish the same purpose, that there could not possibly be any conflict between them, and that they were not enemies but friends.

The medical journals of standing from one end of the country to the other, he affirmed, were rendering great aid to the cause of reform in medical education, and the times were propitious.

He concluded by urging united effort by the friends of medical education, saying that "the reproach cast upon us through a refusal to recognize our diplomas in Europe cannot be overcome until we rise in our might and wage a relentless war against ignorance, that shall not cease until an American State license is recognized as a passport to good professional standing in every civilized country in the world."

---

### MEDICAL EXPERT TESTIMONY.

A committee of the Medical Society of the State of New York, appointed to report upon the most feasible plan by which present methods of introducing medical expert testimony can be improved, submitted the following:

WHEREAS, The present method of obtaining medical expert testimony tends to lessen the value of such testimony and to bring the medical profession into disrepute; therefore, be it

*Resolved*, That the medical society of the State of New York would recommend the enactment of a law by the legislature providing for the appointment of experts by the courts, and that only physicians of repute in the particular branch of medical science to which the question calling for expert opinion relates, shall be appointed; that the function of the experts so appointed shall be advisory, and the number thus appointed shall be such as to adequately represent the court, and both sides of the question at issue, as in the judgment of the court shall seem necessary; that the experts so appointed shall have full and free access to all the evidence in the case, as well as access to the plaintiff or defendant in person as the case may be, if the issue involves his mental or physical state. That the expert shall submit to the court for transmission to the jury a report in writing, setting forth their conclusion and the facts in evidence on which such conclusion is based; that the cross-examination of such experts shall be limited to the facts and opinions embraced in their testimony as embodied in their report, and that their compensation shall be fixed by the court at a rate that is reasonable for professional services of such a nature.

